# Dimethyl 2-butyl-2-(3,5-di-*tert*-butyl-4-hydroxy­benz­yl)malonate

**DOI:** 10.1107/S1600536809035697

**Published:** 2009-09-12

**Authors:** Tao Zeng, Wan-Zhong Ren

**Affiliations:** a Chemistry & Biology College, Yantai University, Yantai 264005, People’s Republic of China

## Abstract

The title compound, C_25_H_38_O_5_, was formed by the reaction of dimethyl 2-butyl­malonate and 2,6-di-*tert*-butyl-4-[(dimethyl­amino)meth­yl]phenol. In the crystal structure, mol­ecules are linked by inter­molecular O—H⋯O hydrogen bonds into chains along [010].

## Related literature

For background to hindered phenols and hindered amines, see: Denisov (1991 ▶); Klemchuk & Gande (1998 ▶); Yamazaki & Seguchi (1997 ▶); Rasberger (1980 ▶); Eggensperger *et al.* (1974 ▶, 1976 ▶). For a related structure, see: Zeng & Chen (2006 ▶).
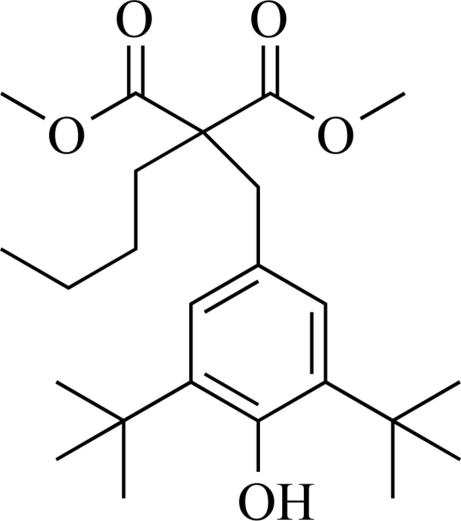


## Experimental

### 

#### Crystal data

C_24_H_38_O_5_
*M* *_r_* = 406.54
Monoclinic, 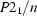

*a* = 10.854 (2) Å
*b* = 10.341 (2) Å
*c* = 22.899 (5) Å
β = 98.838 (4)°
*V* = 2539.7 (9) Å^3^
*Z* = 4
Mo *K*α radiation
μ = 0.07 mm^−1^
*T* = 294 K
0.24 × 0.20 × 0.16 mm

#### Data collection

Bruker SMART CCD diffractometer
Absorption correction: multi-scan (*SADABS*; Sheldrick, 1996 ▶) *T*_min_ = 0.983, *T*_max_ = 0.988
12778 measured reflections
4475 independent reflections
2448 reflections with *I* > 2σ(*I*)
*R*_int_ = 0.048

#### Refinement

*R*[*F* ^2^ > 2σ(*F* ^2^)] = 0.048
*wR*(*F* ^2^) = 0.142
*S* = 1.01
4475 reflections
272 parameters
12 restraints
H-atom parameters constrained
Δρ_max_ = 0.16 e Å^−3^
Δρ_min_ = −0.18 e Å^−3^

### 

Data collection: *SMART* (Bruker, 1997 ▶); cell refinement: *SAINT* (Bruker, 1997 ▶); data reduction: *SAINT*; program(s) used to solve structure: *SHELXS97* (Sheldrick, 2008 ▶); program(s) used to refine structure: *SHELXL97* (Sheldrick, 2008 ▶); molecular graphics: *SHELXTL* (Sheldrick, 2008 ▶) and *PLATON* (Spek, 2009 ▶); software used to prepare material for publication: *SHELXTL* (Sheldrick, 2008 ▶).

## Supplementary Material

Crystal structure: contains datablocks I, global. DOI: 10.1107/S1600536809035697/lh2893sup1.cif

Structure factors: contains datablocks I. DOI: 10.1107/S1600536809035697/lh2893Isup2.hkl

Additional supplementary materials:  crystallographic information; 3D view; checkCIF report

## Figures and Tables

**Table 1:** Hydrogen-bond geometry (Å, °)

*D*—H⋯*A*	*D*—H	H⋯*A*	*D*⋯*A*	*D*—H⋯*A*
O1—H1⋯O2^i^	0.82	2.23	2.832 (2)	130

Symmetry code: (i) 
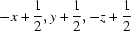
.

**Fig. 1.**
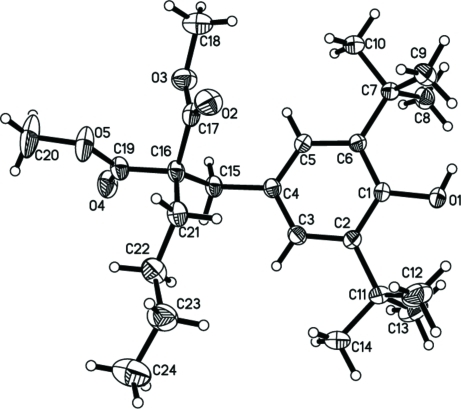
The molecular structure of (I). Displacement ellipsoids are drawn at the 30% probability level and H atoms are shown as small spheres of arbitrary radii.

**Fig. 2.**
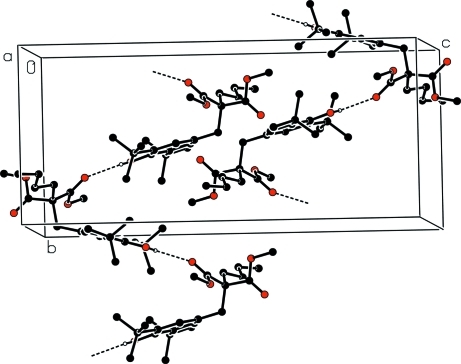
Part of the crystal structure of (I) with dashed lines indicating O—H···O hydrogen bonds. Only H atoms involved in hydrogen bonds are shown.

## supplementary crystallographic information

### Comment

Hindered phenols are widely used as antioxidants while hindered amines are used as light stabilizers in polymers and lubricants because of their special structures (Denisov, 1991; Klemchuk & Gande, 1998; Yamazaki & Seguchi, 1997). In a former paper (Zeng & Chen, 2006), we reported the crystal structure of bis(1,2,2,6,6-pentamethylpiperidin-4-yl)butylmalonate, a key intermediate in the peparation of 'Tinuvin 144' which is a light stabilizer of the hindered amine class that also contains an oxidant unit of the sterically hindered phenol type (Rasberger, 1980; Eggensperger *et al.*, 1974;1976). The title compound was prepared as an intermediate when we attempted to synthesize 'Tinuvin 144' by a different route.

The molecular structure of the title compound (I) is shown in Fig. 1. The phenolic hydroxyl group is sterically hindered by the adjacent bulky *tert*-butyl groups as is indicated by a shorter than normal H···H contact [H1···H9B = 1.87Å]. In the crystal structure, molecules are linked by intermolecular O-H···O hydrogen bonds into one-dimensional chains along [010] (see Fig. 2).

### Experimental

A mixture of bis(1,2,2,6,6-pentamethylpiperidin-4-yl) 2-butylmalonate (11.67 g,0.025 mol) and 2,6-di-*tert*-butyl-4-((dimethylamino)methyl)phenol (6.59 g, 0.025 mol)was dissloved in toluene (100 ml), stirred and heated to reflux. Then 0.2 g lithium amide was added and stirred for a further 4 h, followed by extraction with ether (30 ml) and drying with anhydrous magnesium sulfate. The solvent was removed by vacuum evaporation at 318 K, and the product was filtered and washed with methanol (10 ml). The title compound (I) (15.05 g) was obtained in 87.9% yield. Suitable crystals (m.p. 420–422 K) were obtained by slow evaporation of a solution of (I) in a mixture of THF and methanol.

### Refinement

The H atom of the O—H group was initially located in a difference Fourier map but subsequently included in a calculated position O—H = 0.82 Å and *U*_iso_(H) = 1.5*U*_eq_(O). All other H atoms were positioned geometrically with C-H distances in the range 0.93–0.97 Å, and they were refined using a riding-model approximation  with *U*_iso_(H) = 1.2*U*_eq_(C) or 1.5*U*_eq_(C) for methyl H atoms.

### Crystal data

**Table:**

C_24_H_38_O_5_	*F*(000) = 888
*M**_r_* = 406.54	*D*_x_ = 1.063 Mg m^−^^3^
Monoclinic, *P*2_1_/*n*	Mo *K*α radiation, λ = 0.71073 Å
Hall symbol: -P 2yn	Cell parameters from 2194 reflections
*a* = 10.854 (2) Å	θ = 2.2–21.1°
*b* = 10.341 (2) Å	µ = 0.07 mm^−^^1^
*c* = 22.899 (5) Å	*T* = 294 K
β = 98.838 (4)°	Block, colourless
*V* = 2539.7 (9) Å^3^	0.24 × 0.20 × 0.16 mm
*Z* = 4	

### Data collection

**Table:**

Bruker SMART CCD diffractometer	4475 independent reflections
Radiation source: fine-focus sealed tube	2448 reflections with *I* > 2σ(*I*)
graphite	*R*_int_ = 0.048
φ and ω scans	θ_max_ = 25.0°, θ_min_ = 1.8°
Absorption correction: multi-scan (*SADABS*; Sheldrick, 1996)	*h* = −11→12
*T*_min_ = 0.983, *T*_max_ = 0.988	*k* = −12→11
12778 measured reflections	*l* = −27→21

### Refinement

**Table:**

Refinement on *F*^2^	Primary atom site location: structure-invariant direct methods
Least-squares matrix: full	Secondary atom site location: difference Fourier map
*R*[*F*^2^ > 2σ(*F*^2^)] = 0.048	Hydrogen site location: inferred from neighbouring sites
*wR*(*F*^2^) = 0.142	H-atom parameters constrained
*S* = 1.01	*w* = 1/[σ^2^(*F*_o_^2^) + (0.0654*P*)^2^ + 0.1568*P*] where *P* = (*F*_o_^2^ + 2*F*_c_^2^)/3
4475 reflections	(Δ/σ)_max_ < 0.001
272 parameters	Δρ_max_ = 0.16 e Å^−^^3^
12 restraints	Δρ_min_ = −0.18 e Å^−^^3^

### Special details

**Table:**

Geometry. All e.s.d.'s (except the e.s.d. in the dihedral angle between two l.s. planes) are estimated using the full covariance matrix. The cell e.s.d.'s are taken into account individually in the estimation of e.s.d.'s in distances, angles and torsion angles; correlations between e.s.d.'s in cell parameters are only used when they are defined by crystal symmetry. An approximate (isotropic) treatment of cell e.s.d.'s is used for estimating e.s.d.'s involving l.s. planes.
Refinement. Refinement of *F*^2^ against ALL reflections. The weighted *R*-factor *wR* and goodness of fit *S* are based on *F*^2^, conventional *R*-factors *R* are based on *F*, with *F* set to zero for negative *F*^2^. The threshold expression of *F*^2^ > σ(*F*^2^) is used only for calculating *R*-factors(gt) *etc*. and is not relevant to the choice of reflections for refinement. *R*-factors based on *F*^2^ are statistically about twice as large as those based on *F*, and *R*- factors based on ALL data will be even larger.

### Fractional atomic coordinates and isotropic or equivalent isotropic displacement parameters (Å^2^)

**Table:**

	*x*	*y*	*z*	*U*_iso_*/*U*_eq_	
O1	0.41570 (13)	1.09610 (18)	0.27640 (6)	0.0596 (5)	
H1	0.3622	1.1119	0.2971	0.089*	
O2	0.11836 (16)	0.68114 (18)	0.10961 (7)	0.0672 (5)	
O3	−0.01613 (15)	0.81627 (17)	0.05786 (7)	0.0607 (5)	
O4	0.11420 (16)	0.87050 (17)	−0.07043 (7)	0.0645 (5)	
O5	0.07880 (19)	0.67112 (18)	−0.04056 (7)	0.0780 (6)	
C1	0.35939 (19)	1.0593 (2)	0.22102 (8)	0.0374 (5)	
C2	0.44117 (18)	1.0357 (2)	0.18030 (9)	0.0364 (5)	
C3	0.3872 (2)	1.0021 (2)	0.12350 (9)	0.0400 (5)	
H3	0.4393	0.9863	0.0956	0.048*	
C4	0.25997 (19)	0.9908 (2)	0.10623 (8)	0.0366 (5)	
C5	0.18367 (19)	1.01343 (19)	0.14827 (8)	0.0362 (5)	
H5	0.0978	1.0068	0.1371	0.043*	
C6	0.22977 (18)	1.04564 (19)	0.20643 (9)	0.0344 (5)	
C7	0.14008 (18)	1.0678 (2)	0.25207 (9)	0.0397 (5)	
C8	0.1437 (2)	1.2092 (2)	0.27215 (10)	0.0550 (7)	
H8A	0.1170	1.2639	0.2388	0.082*	
H8B	0.2273	1.2317	0.2893	0.082*	
H8C	0.0892	1.2205	0.3010	0.082*	
C9	0.1722 (2)	0.9759 (2)	0.30526 (10)	0.0564 (7)	
H9A	0.1136	0.9882	0.3322	0.085*	
H9B	0.2548	0.9941	0.3251	0.085*	
H9C	0.1681	0.8880	0.2916	0.085*	
C10	0.0049 (2)	1.0388 (3)	0.22531 (10)	0.0609 (7)	
H10A	−0.0481	1.0514	0.2548	0.091*	
H10B	−0.0016	0.9509	0.2118	0.091*	
H10C	−0.0205	1.0959	0.1926	0.091*	
C11	0.58343 (19)	1.0484 (2)	0.19695 (10)	0.0456 (6)	
C12	0.6333 (2)	0.9627 (3)	0.24987 (11)	0.0730 (8)	
H12A	0.7217	0.9746	0.2599	0.109*	
H12B	0.6158	0.8737	0.2399	0.109*	
H12C	0.5936	0.9861	0.2830	0.109*	
C13	0.6192 (2)	1.1891 (3)	0.21131 (13)	0.0798 (9)	
H13A	0.5885	1.2427	0.1780	0.120*	
H13B	0.7083	1.1964	0.2199	0.120*	
H13C	0.5831	1.2166	0.2450	0.120*	
C14	0.6513 (2)	1.0060 (3)	0.14653 (11)	0.0823 (10)	
H14A	0.6255	1.0594	0.1126	0.123*	
H14B	0.6314	0.9174	0.1368	0.123*	
H14C	0.7396	1.0145	0.1586	0.123*	
C15	0.2057 (2)	0.9562 (2)	0.04293 (8)	0.0418 (6)	
H15A	0.2616	0.9878	0.0169	0.050*	
H15B	0.1268	1.0008	0.0326	0.050*	
C16	0.1842 (2)	0.8100 (2)	0.03162 (8)	0.0404 (6)	
C17	0.0948 (2)	0.7595 (2)	0.07120 (10)	0.0454 (6)	
C18	−0.1102 (3)	0.7799 (3)	0.09247 (13)	0.0861 (10)	
H18A	−0.1429	0.6965	0.0800	0.129*	
H18B	−0.1762	0.8426	0.0871	0.129*	
H18C	−0.0743	0.7765	0.1335	0.129*	
C19	0.1224 (2)	0.7911 (2)	−0.03234 (10)	0.0485 (6)	
C20	0.0126 (4)	0.6420 (3)	−0.09909 (12)	0.1183 (14)	
H20A	−0.0592	0.6970	−0.1075	0.177*	
H20B	−0.0136	0.5532	−0.1006	0.177*	
H20C	0.0667	0.6565	−0.1279	0.177*	
C21	0.3054 (2)	0.7306 (2)	0.04303 (10)	0.0499 (6)	
H21A	0.3474	0.7503	0.0825	0.060*	
H21B	0.2835	0.6396	0.0423	0.060*	
C22	0.3966 (2)	0.7522 (3)	0.00000 (11)	0.0655 (7)	
H22A	0.3594	0.7213	−0.0387	0.079*	
H22B	0.4109	0.8444	−0.0032	0.079*	
C23	0.5202 (3)	0.6857 (3)	0.01765 (13)	0.0813 (9)	
H23A	0.5048	0.5961	0.0269	0.098*	
H23B	0.5626	0.7259	0.0534	0.098*	
C24	0.6056 (3)	0.6890 (3)	−0.02852 (16)	0.1106 (12)	
H24A	0.5647	0.6494	−0.0642	0.166*	
H24B	0.6808	0.6425	−0.0143	0.166*	
H24C	0.6257	0.7771	−0.0364	0.166*	

### Atomic displacement parameters (Å^2^)

**Table:**

	*U*^11^	*U*^22^	*U*^33^	*U*^12^	*U*^13^	*U*^23^
O1	0.0364 (9)	0.1016 (14)	0.0393 (9)	−0.0034 (9)	0.0012 (7)	−0.0246 (9)
O2	0.0723 (13)	0.0773 (13)	0.0512 (11)	−0.0060 (10)	0.0072 (9)	0.0247 (10)
O3	0.0506 (11)	0.0706 (12)	0.0615 (11)	0.0020 (9)	0.0102 (9)	0.0068 (9)
O4	0.0879 (14)	0.0687 (13)	0.0331 (9)	−0.0081 (10)	−0.0027 (9)	0.0055 (9)
O5	0.1241 (17)	0.0569 (13)	0.0447 (10)	−0.0224 (11)	−0.0135 (10)	−0.0085 (8)
C1	0.0365 (13)	0.0437 (14)	0.0309 (12)	−0.0030 (10)	0.0018 (10)	−0.0057 (10)
C2	0.0344 (12)	0.0369 (13)	0.0373 (13)	−0.0036 (10)	0.0040 (10)	−0.0016 (10)
C3	0.0431 (14)	0.0418 (14)	0.0366 (13)	−0.0012 (10)	0.0107 (11)	−0.0020 (10)
C4	0.0412 (13)	0.0359 (13)	0.0326 (12)	0.0011 (10)	0.0050 (10)	0.0016 (10)
C5	0.0345 (12)	0.0369 (13)	0.0357 (12)	−0.0021 (10)	0.0011 (10)	−0.0015 (10)
C6	0.0342 (13)	0.0347 (13)	0.0346 (12)	−0.0002 (9)	0.0057 (10)	−0.0029 (9)
C7	0.0329 (12)	0.0488 (15)	0.0377 (12)	−0.0001 (10)	0.0065 (10)	−0.0055 (11)
C8	0.0520 (16)	0.0604 (18)	0.0524 (15)	0.0115 (12)	0.0077 (12)	−0.0132 (12)
C9	0.0606 (16)	0.0639 (18)	0.0474 (14)	−0.0046 (13)	0.0171 (12)	−0.0003 (12)
C10	0.0394 (14)	0.089 (2)	0.0565 (16)	−0.0098 (13)	0.0135 (12)	−0.0126 (14)
C11	0.0326 (13)	0.0545 (16)	0.0501 (14)	−0.0056 (11)	0.0082 (11)	−0.0040 (11)
C12	0.0417 (16)	0.089 (2)	0.0850 (19)	0.0067 (14)	0.0009 (14)	0.0169 (16)
C13	0.0532 (18)	0.070 (2)	0.115 (2)	−0.0186 (14)	0.0079 (17)	−0.0108 (17)
C14	0.0392 (16)	0.136 (3)	0.0758 (19)	−0.0085 (16)	0.0207 (14)	−0.0216 (19)
C15	0.0512 (14)	0.0433 (14)	0.0294 (12)	−0.0009 (11)	0.0022 (10)	0.0007 (10)
C16	0.0520 (15)	0.0435 (14)	0.0245 (11)	−0.0007 (11)	0.0024 (10)	0.0021 (10)
C17	0.0540 (16)	0.0446 (15)	0.0348 (13)	−0.0055 (12)	−0.0015 (12)	−0.0046 (11)
C18	0.0618 (19)	0.108 (3)	0.095 (2)	−0.0141 (17)	0.0302 (17)	−0.0006 (19)
C19	0.0607 (17)	0.0496 (17)	0.0345 (14)	−0.0012 (13)	0.0048 (12)	−0.0024 (12)
C20	0.180 (4)	0.099 (3)	0.0592 (19)	−0.035 (2)	−0.034 (2)	−0.0227 (18)
C21	0.0611 (16)	0.0472 (15)	0.0400 (13)	0.0031 (12)	0.0034 (12)	−0.0008 (11)
C22	0.0691 (19)	0.0700 (19)	0.0591 (17)	0.0084 (15)	0.0154 (15)	−0.0021 (14)
C23	0.075 (2)	0.074 (2)	0.099 (2)	0.0108 (16)	0.0258 (18)	−0.0095 (17)
C24	0.093 (3)	0.108 (3)	0.142 (3)	0.013 (2)	0.052 (2)	0.002 (2)

### Geometric parameters (Å, °)

**Table:**

O1—C1	1.374 (2)	C12—H12A	0.9600
O1—H1	0.8200	C12—H12B	0.9600
O2—C17	1.195 (3)	C12—H12C	0.9600
O3—C17	1.332 (3)	C13—H13A	0.9600
O3—C18	1.435 (3)	C13—H13B	0.9600
O4—C19	1.191 (3)	C13—H13C	0.9600
O5—C19	1.331 (3)	C14—H14A	0.9600
O5—C20	1.452 (3)	C14—H14B	0.9600
C1—C6	1.402 (3)	C14—H14C	0.9600
C1—C2	1.404 (3)	C15—C16	1.546 (3)
C2—C3	1.386 (3)	C15—H15A	0.9700
C2—C11	1.538 (3)	C15—H15B	0.9700
C3—C4	1.382 (3)	C16—C17	1.518 (3)
C3—H3	0.9300	C16—C19	1.527 (3)
C4—C5	1.383 (3)	C16—C21	1.539 (3)
C4—C15	1.521 (3)	C18—H18A	0.9600
C5—C6	1.389 (3)	C18—H18B	0.9600
C5—H5	0.9300	C18—H18C	0.9600
C6—C7	1.551 (3)	C20—H20A	0.9600
C7—C8	1.531 (3)	C20—H20B	0.9600
C7—C10	1.531 (3)	C20—H20C	0.9600
C7—C9	1.543 (3)	C21—C22	1.517 (3)
C8—H8A	0.9600	C21—H21A	0.9700
C8—H8B	0.9600	C21—H21B	0.9700
C8—H8C	0.9600	C22—C23	1.507 (3)
C9—H9A	0.9600	C22—H22A	0.9700
C9—H9B	0.9600	C22—H22B	0.9700
C9—H9C	0.9600	C23—C24	1.510 (3)
C10—H10A	0.9600	C23—H23A	0.9700
C10—H10B	0.9600	C23—H23B	0.9700
C10—H10C	0.9600	C24—H24A	0.9600
C11—C14	1.526 (3)	C24—H24B	0.9600
C11—C13	1.528 (3)	C24—H24C	0.9600
C11—C12	1.532 (3)		
			
C1—O1—H1	109.5	H13B—C13—H13C	109.5
C17—O3—C18	116.9 (2)	C11—C14—H14A	109.5
C19—O5—C20	116.0 (2)	C11—C14—H14B	109.5
O1—C1—C6	122.37 (18)	H14A—C14—H14B	109.5
O1—C1—C2	115.09 (18)	C11—C14—H14C	109.5
C6—C1—C2	122.54 (18)	H14A—C14—H14C	109.5
C3—C2—C1	116.58 (19)	H14B—C14—H14C	109.5
C3—C2—C11	121.17 (19)	C4—C15—C16	114.60 (16)
C1—C2—C11	122.24 (18)	C4—C15—H15A	108.6
C4—C3—C2	123.3 (2)	C16—C15—H15A	108.6
C4—C3—H3	118.3	C4—C15—H15B	108.6
C2—C3—H3	118.3	C16—C15—H15B	108.6
C3—C4—C5	117.74 (18)	H15A—C15—H15B	107.6
C3—C4—C15	121.09 (18)	C17—C16—C19	107.60 (18)
C5—C4—C15	121.17 (19)	C17—C16—C21	108.89 (18)
C4—C5—C6	122.81 (19)	C19—C16—C21	109.42 (18)
C4—C5—H5	118.6	C17—C16—C15	109.27 (18)
C6—C5—H5	118.6	C19—C16—C15	108.57 (17)
C5—C6—C1	116.93 (18)	C21—C16—C15	112.95 (18)
C5—C6—C7	120.70 (18)	O2—C17—O3	123.5 (2)
C1—C6—C7	122.36 (17)	O2—C17—C16	126.0 (2)
C8—C7—C10	106.65 (18)	O3—C17—C16	110.5 (2)
C8—C7—C9	111.05 (18)	O3—C18—H18A	109.5
C10—C7—C9	106.34 (18)	O3—C18—H18B	109.5
C8—C7—C6	110.73 (17)	H18A—C18—H18B	109.5
C10—C7—C6	111.32 (16)	O3—C18—H18C	109.5
C9—C7—C6	110.59 (17)	H18A—C18—H18C	109.5
C7—C8—H8A	109.5	H18B—C18—H18C	109.5
C7—C8—H8B	109.5	O4—C19—O5	123.6 (2)
H8A—C8—H8B	109.5	O4—C19—C16	125.9 (2)
C7—C8—H8C	109.5	O5—C19—C16	110.4 (2)
H8A—C8—H8C	109.5	O5—C20—H20A	109.5
H8B—C8—H8C	109.5	O5—C20—H20B	109.5
C7—C9—H9A	109.5	H20A—C20—H20B	109.5
C7—C9—H9B	109.5	O5—C20—H20C	109.5
H9A—C9—H9B	109.5	H20A—C20—H20C	109.5
C7—C9—H9C	109.5	H20B—C20—H20C	109.5
H9A—C9—H9C	109.5	C22—C21—C16	115.95 (19)
H9B—C9—H9C	109.5	C22—C21—H21A	108.3
C7—C10—H10A	109.5	C16—C21—H21A	108.3
C7—C10—H10B	109.5	C22—C21—H21B	108.3
H10A—C10—H10B	109.5	C16—C21—H21B	108.3
C7—C10—H10C	109.5	H21A—C21—H21B	107.4
H10A—C10—H10C	109.5	C23—C22—C21	113.7 (2)
H10B—C10—H10C	109.5	C23—C22—H22A	108.8
C14—C11—C13	107.5 (2)	C21—C22—H22A	108.8
C14—C11—C12	106.2 (2)	C23—C22—H22B	108.8
C13—C11—C12	109.5 (2)	C21—C22—H22B	108.8
C14—C11—C2	111.75 (18)	H22A—C22—H22B	107.7
C13—C11—C2	110.30 (19)	C22—C23—C24	114.8 (3)
C12—C11—C2	111.46 (18)	C22—C23—H23A	108.6
C11—C12—H12A	109.5	C24—C23—H23A	108.6
C11—C12—H12B	109.5	C22—C23—H23B	108.6
H12A—C12—H12B	109.5	C24—C23—H23B	108.6
C11—C12—H12C	109.5	H23A—C23—H23B	107.5
H12A—C12—H12C	109.5	C23—C24—H24A	109.5
H12B—C12—H12C	109.5	C23—C24—H24B	109.5
C11—C13—H13A	109.5	H24A—C24—H24B	109.5
C11—C13—H13B	109.5	C23—C24—H24C	109.5
H13A—C13—H13B	109.5	H24A—C24—H24C	109.5
C11—C13—H13C	109.5	H24B—C24—H24C	109.5
H13A—C13—H13C	109.5		
			
O1—C1—C2—C3	−178.16 (19)	C1—C2—C11—C12	56.3 (3)
C6—C1—C2—C3	2.2 (3)	C3—C4—C15—C16	92.5 (2)
O1—C1—C2—C11	0.8 (3)	C5—C4—C15—C16	−88.2 (2)
C6—C1—C2—C11	−178.84 (19)	C4—C15—C16—C17	59.5 (2)
C1—C2—C3—C4	−0.3 (3)	C4—C15—C16—C19	176.56 (18)
C11—C2—C3—C4	−179.3 (2)	C4—C15—C16—C21	−61.9 (2)
C2—C3—C4—C5	−0.6 (3)	C18—O3—C17—O2	0.4 (3)
C2—C3—C4—C15	178.8 (2)	C18—O3—C17—C16	−178.90 (19)
C3—C4—C5—C6	−0.5 (3)	C19—C16—C17—O2	126.1 (2)
C15—C4—C5—C6	−179.80 (19)	C21—C16—C17—O2	7.6 (3)
C4—C5—C6—C1	2.2 (3)	C15—C16—C17—O2	−116.2 (3)
C4—C5—C6—C7	−179.01 (19)	C19—C16—C17—O3	−54.6 (2)
O1—C1—C6—C5	177.25 (19)	C21—C16—C17—O3	−173.07 (18)
C2—C1—C6—C5	−3.1 (3)	C15—C16—C17—O3	63.1 (2)
O1—C1—C6—C7	−1.5 (3)	C20—O5—C19—O4	−3.3 (4)
C2—C1—C6—C7	178.14 (19)	C20—O5—C19—C16	177.3 (2)
C5—C6—C7—C8	−113.8 (2)	C17—C16—C19—O4	131.7 (3)
C1—C6—C7—C8	64.8 (2)	C21—C16—C19—O4	−110.1 (3)
C5—C6—C7—C10	4.6 (3)	C15—C16—C19—O4	13.6 (3)
C1—C6—C7—C10	−176.7 (2)	C17—C16—C19—O5	−48.9 (3)
C5—C6—C7—C9	122.6 (2)	C21—C16—C19—O5	69.3 (2)
C1—C6—C7—C9	−58.7 (3)	C15—C16—C19—O5	−167.05 (19)
C3—C2—C11—C14	−6.2 (3)	C17—C16—C21—C22	169.91 (19)
C1—C2—C11—C14	174.9 (2)	C19—C16—C21—C22	52.6 (3)
C3—C2—C11—C13	113.3 (2)	C15—C16—C21—C22	−68.5 (2)
C1—C2—C11—C13	−65.6 (3)	C16—C21—C22—C23	172.5 (2)
C3—C2—C11—C12	−124.8 (2)	C21—C22—C23—C24	171.4 (2)

### Hydrogen-bond geometry (Å, °)

**Table:**

*D*—H···*A*	*D*—H	H···*A*	*D*···*A*	*D*—H···*A*
O1—H1···O2^i^	0.82	2.23	2.832 (2)	130

Symmetry codes: (i) −*x*+1/2, *y*+1/2, −*z*+1/2.
